# Screening and characterization of long noncoding RNAs involved in the albinism of *Ananas comosus* var. *bracteatus* leaves

**DOI:** 10.1371/journal.pone.0225602

**Published:** 2019-11-22

**Authors:** Zhen Lin, Yingyuan Xiong, Yanbin Xue, Meiqin Mao, Yixuan Xiang, Yehua He, Fatima Rafique, Hao Hu, Jiawen Liu, Xi Li, Lingxia Sun, Zhuo Huang, Jun Ma

**Affiliations:** 1 College of Landscape Architecture of Sichuan Agricultural University, Chengdu, Sichuan, China; 2 Horticultural Biotechnology College of South China Agricultural University, Guangzhou, Guangdong, China; ICAR-Indian Institute of Agricultural Biotechnology, INDIA

## Abstract

Long noncoding RNAs (lncRNAs) have been reported to play key regulatory roles in plant growth, development, and biotic and abiotic stress physiology. Revealing the mechanism of lncRNA regulation in the albino portions of leaves is important for understanding the development of chimeric leaves in *Ananas comosus* var. *bracteatus*. In this study, a total of 3,543 candidate lncRNAs were identified, among which 1,451 were differentially expressed between completely green (CGr) and completely white (CWh) leaves. LncRNAs tend to have shorter transcripts, lower expression levels, and greater expression specificity than protein-coding genes. Predicted lncRNA targets were functionally annotated by the Gene Ontology (GO), Clusters of Orthologous Groups (COG) and Kyoto Encyclopedia of Genes and Genomes (KEGG) databases. A lncRNA-mRNA interaction network was constructed, and 36 target mRNAs related to chlorophyll metabolism were predicted to interact with 86 lncRNAs. Among these, 25 significantly differentially expressed lncRNAs putatively interacted with 16 target mRNAs. Based on an expression pattern analysis of the lncRNAs and their target mRNAs, the lncRNAs targeting magnesium chelatase subunit H (ChlH), protochlorophyllide oxidoreductase (POR), and heme o synthase (COX10) were suggested as key regulators of chlorophyll metabolism. This study provides the first lncRNA database for *A*. *comosus* var. *bracteatus* and contributes greatly to understanding the mechanism of epigenetic regulation of leaf albinism.

## Introduction

Noncoding RNAs (ncRNAs) are RNA transcripts that do not encode a protein. In the past decade, a great diversity of ncRNAs has been observed. General conventions divide ncRNAs into two main categories: small ncRNAs (<200 bp) and long noncoding RNAs (lncRNAs; >200 bp) [1]. LncRNAs are defined as RNAs that are at least 200 nucleotides (nt) in length, are independently transcribed, and bear a molecular resemblance to mRNAs but do not have recognizable potential to encode functional proteins. The 200 nt cutoff excludes most canonical ncRNAs, such as small nucleolar RNAs (snoRNAs), small nuclear RNAs (snRNAs) and tRNAs, and roughly corresponds to the retention threshold of protocols for the purification of long RNAs [2]. Analyses of lncRNAs remain scarce, despite growing interest in these genes. Recent studies have identified thousands of lncRNAs in the human, mouse, fruit fly, nematode and zebrafish genomes [3].

Recent technological advances, such as tiling arrays and RNA deep sequencing (RNA-seq), have made it possible to survey the transcriptomes of many organisms to an unprecedented degree [4]. LncRNAs have gained widespread attention in recent years as a potentially new and crucial mode of biological regulation [5]. LncRNAs are strikingly similar to mRNAs; in animals, these RNAs are RNA polymerase II transcripts that are capped, spliced and polyadenylated but do not function as templates for protein synthesis [6]. However, lncRNAs can be transcribed by polymerases II, IV, and V; therefore, some lncRNAs in plants may lack poly-A tails [7]. Recent studies have shown that lncRNAs can be folded into complex secondary and higher structures that provide increased potential and versatility for identifying proteins and targets [8,9]. Therefore, lncRNAs may regulate the expression of protein-coding genes at the posttranscriptional and transcriptional levels [10]. An increasing number of studies have shown that lncRNAs play a crucial role in epigenetic regulation [11,12], for example, in cell proliferation, differentiation and individual development in animals [13–16]. The involvement of lncRNAs in human diseases could be far more prevalent than previously appreciated [17]. In plants, lncRNAs are differentially expressed in various organs and under different treatment conditions, which indicates that lncRNAs can modulate gene activity during development and in response to external stimuli [18]. In *Arabidopsis*, rosette flowering time was significantly delayed, and leaf diameter was significantly reduced by the lncRNA *Npc48* [19,20]. The rice-specific lncRNA *LDMAR* was found to be a key gene for controlling photoperiod-sensitive male sterility (PSMS) [21]. Overexpression of the pollen-specific lncRNA *Zm401* resulted in anther degradation and, thus, seriously affected pollen development [22].

Pineapple is a perennial, herbaceous monocot of the family Bromeliaceae, subfamily Bromelioideae, in the order Poales. According to the recent classification, Coppens d’Eeckenbrugge and Leal proposed one genus, *Ananas*, with two species, *Ananas comosus* (L.) Merr. (diploid, 2n = 2x = 50) and *A*. *macrodontes* É.Morren (tetraploid, 2n = 4x = 100). *A*. *comosus* is subdivided into five botanical varieties: var. *comosus*, var. *ananassoides*, var. *erectifolius*, var. *parguazensis* and var. *bracteatus* [23,24]. The stems and leaves of pineapple crops are widely used as high quality silk fibers in the paper and garment industry [25,26]. As a source of bromelain (a proteolytic enzyme complex), it contains substances and vitamins that are beneficial for human health and is widely used in the pharmaceutical industry [27]. Among the five varieties of *A*. *comosus*, *A*. *comosus* var. *bracteatus* is an important ornamental plant due to its colorful leaves and decorative red fruits. Its decorative leaves consist of normal green cells and albino white cells. *A*. *comosus* var. *bracteatus* is self-incompatible, and thus, tissue culture is a fast and effective method of cultivation of this species. However, the chimeric character is not stable during tissue culture. Only approximately 1% of regenerated plants are chimeric plants, while more than 80% of regenerated plants are completely green (CGr) and completely white (CWh). It is important to understand the mechanisms related to albinism in CWh leaf cells to enhance the stability of this chimeric character [28]. The CGr and CWh plants derived from tissue culture are typical examples of plants with normal green and albino white cells, respectively. Previously, we studied the gene expression patterns of CGr and CWh leaves and found that key genes play important roles in the albinism of CWh leaves [28,29]. However, the mechanism underlying the epigenetic regulation of albinism in the white leaf cells of *A*. *comosus* var. *bracteatus* via lncRNA remains unknown.

In the present study, we used high-throughput RNA-seq to perform genome-wide scanning to identify and characterize lncRNAs in the CWh and CGr leaves of *A*. *comosus* var. *bracteatus* at different developmental stages. A total of 3,543 candidate lncRNAs were identified. Among these lncRNAs, 1,451 were differentially expressed between CGr and CWh leaves during plant development. The target genes of the lncRNAs were annotated with functions in many aspects of leaf metabolism and development, such as functions in the cell component, metabolic process and cellular process categories. Based on this expression pattern analysis of lncRNAs and their target mRNAs, lncRNAs may play important roles in the epigenetic regulation of leaf albinism. These findings provide new insights into the mechanism of lncRNA-based epigenetic regulation of albino leaf cell development in *A*. *comosus* var. *bracteatus*.

## Materials and methods

### Selection of plant material

**Fig 1:**
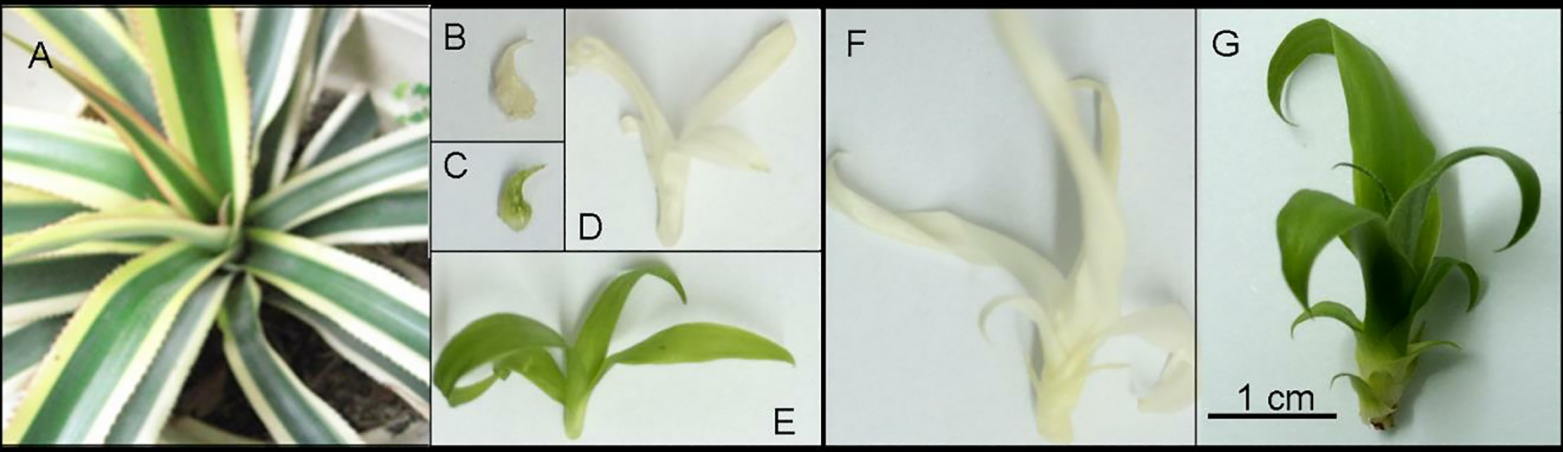
Materials used for lncRNA sequencing. (A) Wild chimeric plant of *A*. *comosus* var. *bracteatus*; (B) Completely white shoots at developmental stage 1 (with unexpanded leaves, WS1); (C) Completely green shoots at developmental stage 1 (GS1); (D) Completely white shoots at developmental stage 2 (with four to five expanded leaves, WS2); (E) Completely green shoots at developmental stage 2 (GS2); (F) Completely white shoots at developmental stage 3 (with ten to twelve expanded leaves, WS3); (G) Completely green shoots at developmental stage 3 (GS3).

Completely green (GS1, GS2, GS3) and completely white (WS1, WS2, WS3) shoots were derived from stem explants of chimeric plants of *A*. *comosus* var. *bracteatus* (Fig 1A) by tissue culture, in accordance with our previous protocol [28]. Three developmental stages were evaluated based on the number of expanded leaves on the shoots [30]. WS1 (Fig 1B) and GS1 (Fig 1C) were completely white and green shoots, respectively, with only unexpanded leaves; WS2 (Fig 1D) and GS2 (Fig 1E) were completely white and green shoots, respectively, with four to five expanded leaves; WS3 (Fig 1F) and GS3 (Fig 1G) were completely white and green shoots, respectively, with ten to twelve expanded leaves. The leaves were collected from at least ten shoots at the above three stages, immediately frozen in liquid nitrogen, and then stored at -80°C until analysis.

### Total RNA extraction and quantification

Total RNA was isolated from six samples using TRIzol (Invitrogen, USA) according to the manufacturer’s instructions. RNA degradation and contamination, especially DNA contamination, was monitored on 1.5% agarose gels. RNA concentration and purity were measured using a NanoDrop 2000 spectrophotometer (Thermo Fisher Scientific, Wilmington, DE). RNA integrity was assessed using the RNA Nano 6000 Assay Kit and the Agilent Bioanalyzer 2100 system (Agilent Technologies, United States of America, CA).

### LncRNA library construction and sequencing

A total amount of 1.5 μg of RNA per sample was used as input material for rRNA removal using the Ribo-Zero rRNA Removal Kit (Epicentre, Madison, WI, USA). Sequencing libraries were generated using the NEBNextR UltraTM Directional RNA Library Prep Kit for IlluminaR (NEB, USA) following the manufacturer’s instructions, and index codes were added to attribute the sequences to each sample. Briefly, fragmentation was carried out using divalent cations at elevated temperatures in NEBNext First-Strand Synthesis Reaction Buffer (5×). First-strand cDNA was synthesized using a random hexamer primer and reverse transcriptase. Second-strand cDNA synthesis was subsequently performed using DNA polymerase I and RNase H. Remaining overhangs were converted into blunt ends via exonuclease/polymerase activities. After adenylation of the 3' ends of the DNA fragments, NEBNext adaptors with a hairpin loop structure were ligated to prepare each sample for hybridization. To preferentially select insert fragments that were 150~200 bp in length, the library fragments were purified with AMPure XP beads (Beckman Coulter, Beverly, USA). Then, 3 μl of USER enzyme (NEB, USA) was incubated with the size-selected, adaptor-ligated cDNA at 37°C for 15 min before PCR. PCR was then performed with Phusion high-fidelity DNA polymerase, universal PCR primers and the index (X) primer. Finally, the PCR products were purified (AMPure XP system), and library quality was assessed on an Agilent Bioanalyzer 2100 and by qPCR. Clustering of the index-coded samples was performed on a cBot cluster generation system using the TruSeq PE Cluster Kit v3-cBot-HS (Illumina) according to the manufacturer’s instructions. After cluster generation, the library preparations were sequenced on an Illumina HiSeq 2500 platform, and paired-end reads were generated (Beijing Biomarker Technologies Co., Ltd., Beijing, China).

### Bioinformatic analysis for identifying lncRNAs

Raw data (raw reads) in fastq format were first processed through in-house Perl scripts. In this step, clean data (clean reads) were obtained by removing adaptors, reads containing more than 10% poly-N sequence, and low-quality bases from the raw data. At the same time, the Q20, Q30, GC content and sequence duplication level of the clean data were calculated. All downstream analyses were based on clean data with high quality. The clean reads were mapped to the reference genome using TopHat2 (v2.0.13) [31]. The transcriptome was assembled using Cufflinks [32] and Scripture based on reads mapped to the reference genome (*A*. *comosus*_321_v3). Alternative splicing was analyzed using ASprofile software (version b-1.0.4) [33]. The assembled transcripts were annotated using the Cuffcompare program from the Cufflinks package. According to Cuffcompare, only transcripts class-coded as “u” (unknown intergenic transcript), “i” (a transfrag falling entirely within a reference intron) and “x” (exonic overlap with reference on the opposite strand) were selected as candidate lncRNAs.

Putative protein-coding RNAs were filtered out using a minimum length and exon number threshold. Transcripts with lengths greater than 200 bp and at least two exons were retained. The CPC, CPAT, CNCI and Pfam tools were utilized to predict the coding ability of transcripts. The intersection of CPC score<0, CPAT score≤0.38, CNCI score<0 and Pfam result “noncoding” were the final newly predicted lncRNAs [34–37]. Different types of lncRNAs, including lincRNAs, intronic lncRNAs, antisense lncRNAs and sense lncRNAs, were selected using Cuffcompare. Cuffdiff (v2.1.1) was used to calculate the fragments per kilobase of transcript per million mapped reads (FPKMs) of both the lncRNAs and coding genes in each sample [38]. Gene FPKMs were computed by summing the FPKMs of the transcripts in each gene group. FPKM was calculated based on the lengths of the fragments and read counts mapped to each fragment.

Based on the mode of lncRNA action on target genes, we used two predictive methods. First, we made predictions based on the positions of the lncRNA and mRNA. LncRNA regulates the expression of neighboring genes, so the protein-coding genes within a range of 100 kb of the lncRNA were defined as potential target genes. Second, we made predictions by base pairing between lncRNA and mRNA. The LncTar [39] target gene prediction tool was used to predict the target gene of each lncRNA. The subject number of raw sequenced data of the lncRNA high-throughput RNA-seq is PRJNA564223 in the NCBI database.

### Differential expression analysis and gene functional annotation

Differential expression analysis of DE-mRNA and DE-lncRNA in the two groups was performed using the EBseq (2010) R package between WS1/GS1, WS2/GS2 and WS3/GS3. The resulting P values were adjusted using the q value [40]. Genes with an adjusted q value<0.01 & | log2(foldchange)|>1 were set as the threshold for significantly differential expression. Gene function was annotated based on the following databases: NCBI nonredundant protein sequences (Nr), Protein family (Pfam), a manually annotated and reviewed protein sequence database (Swiss-Prot), Kyoto Encyclopedia of Genes and Genomes (KEGG), Gene Ontology (GO).

### LncRNA conservation in different species

The full-length sequences of all identified 3,543 *A*. *comosus* var. *bracteatus* lncRNAs were used to blast against the genomes of *Coffea canephora*, *Hordeum vulgare*, *Triticum aestivum*, *Phoenix dactylifera* and *Elaeis guineensis* with word-size = 5 and E-value < 1e^-5^. The coverage value, referred to as the percentage of conserved sequence regions in full-length lncRNAs, was also investigated to predict the species most closely related to *A*. *comosus* var. *bracteatus*.

### GO and KEGG enrichment analysis

GO enrichment analysis of the differentially expressed genes (DEGs) was implemented by the topGO R package [41]. KEGG is a database resource for understanding the high-level functions and utilities of biological systems, such as cells, organisms and ecosystems, at the molecular level, especially for large-scale molecular datasets generated by genome sequencing and other high-throughput experimental technologies. We used KOBAS software to test the statistical enrichment of DEGs in KEGG pathways [42].

### Construction of mRNA–mRNA and lncRNA–mRNA networks

First, the interactions between lncRNAs and mRNAs through targeted relationships were predicted using lncTar (v1.0) with the parameters -d -0.10 and -s F. For the mRNA-mRNA networks, mRNAs were first blasted against the STRING database with E-value 1e^-5^; then, the network of aligned proteins was retrieved from the database; and finally, the interaction network was visualized by Cystoscope [43].

### qRT-PCR identification of lncRNA and potential target gene expression levels

Total RNA from green and white leaves at three developmental stages, as for the sequencing samples, was reverse transcribed using ReverTra Ace® qPCR RT Master Mix (Toyobo) to generate cDNA. qRT-PCR was performed in triplicate using an Applied Biosystems 7900HT (ABI 7500, America) real-time PCR system and the SYBR® Green Real-time PCR Master Mix (Toyobo). The 20-μl reaction system for the PCR contained the following components: 3.4 μl of PCR-grade water, 10 μl of SYBR® Green Real-time PCR Master Mix, 0.8 μl of 10 pmol/μl (10 μM) primer #1, 0.8 μl of 10 pmol/μl (10 μM) primer #2 and 5 μl of template DNA. The cycling conditions for the PCR were as follows: 95°C for 2 min, followed by 40 cycles of 95°C for 15 sec and 58°C for 15 sec. α-Tubulin and 18S rRNA were used as internal controls, and all the primers used are listed in S1 Table. After PCR amplification, a melting curve analysis was carried out to check amplification specificity. Experiments were performed for three biological repeats.

## Results

### Genomic identification of lncRNAs in *Ananas comosus* var. *bracteatus* leaves

**Fig 2:**
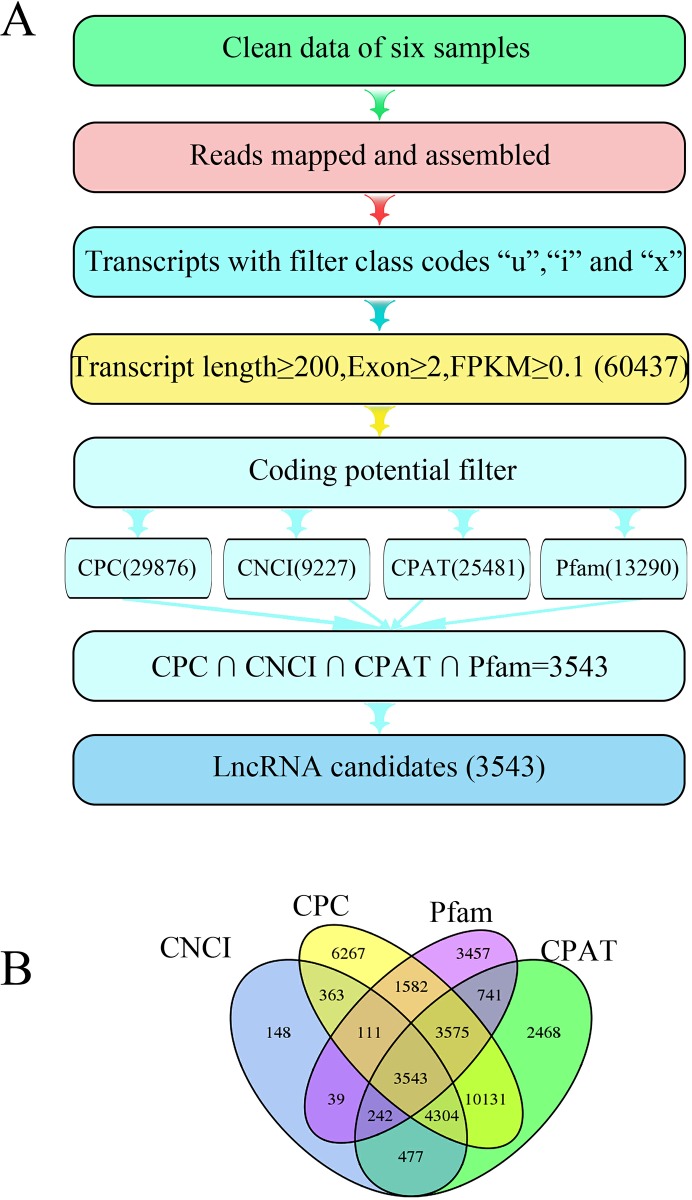
Complete computational pipeline for the systematic identification of lncRNAs in *Ananas comosus* var. *bracteatus* leaves. (A) Detailed schematic diagram of the bioinformatic pipeline for the identification of leaf lncRNAs; (B) Venn diagram of the number of candidate lncRNAs filtered by CPC, CNCI, CPAT and Pfam. CPC: coding potential calculator; CNCI: coding-noncoding index; CPAT: coding potential assessment tool; Pfam: Pfam is a large database of protein families, represented by multiple sequence alignments and hidden Markov models.

In this study, to identify lncRNAs in *A*. *comosus* var. *bracteatus*, high-throughput sequencing of six RNA-seq libraries constructed from CGr and CWh leaves at three developmental stages was performed. Total RNA was isolated, and cDNA libraries were constructed and sequenced using the Illumina HiSeq platform. Approximately 220 million clean reads were obtained from six libraries (Fig 2A). The sequences were mapped to the specified reference genome, *A*.*comosus*_321_v3 (https://phytozome.jgi.doe.gov/pz/portal.html#!info?alias=Org_Acomosus_er), resulting in mapped data. The lncRNAs were identified according to the pipeline shown in Fig 2. The data were filtered using two criteria: (1) length ≥ 200 bp, exon number ≥ 2; (2) FPKM ≥ 0.1 transcripts. As a result, 60,437 transcripts were obtained after filtering. Finally, 3,543 lncRNAs were obtained from the intersection of the CPC, CNCI, CPAT and Pfam results (S2 Table) (Fig 2B).

### Sequence characteristics of the lncRNAs

**Fig 3:**
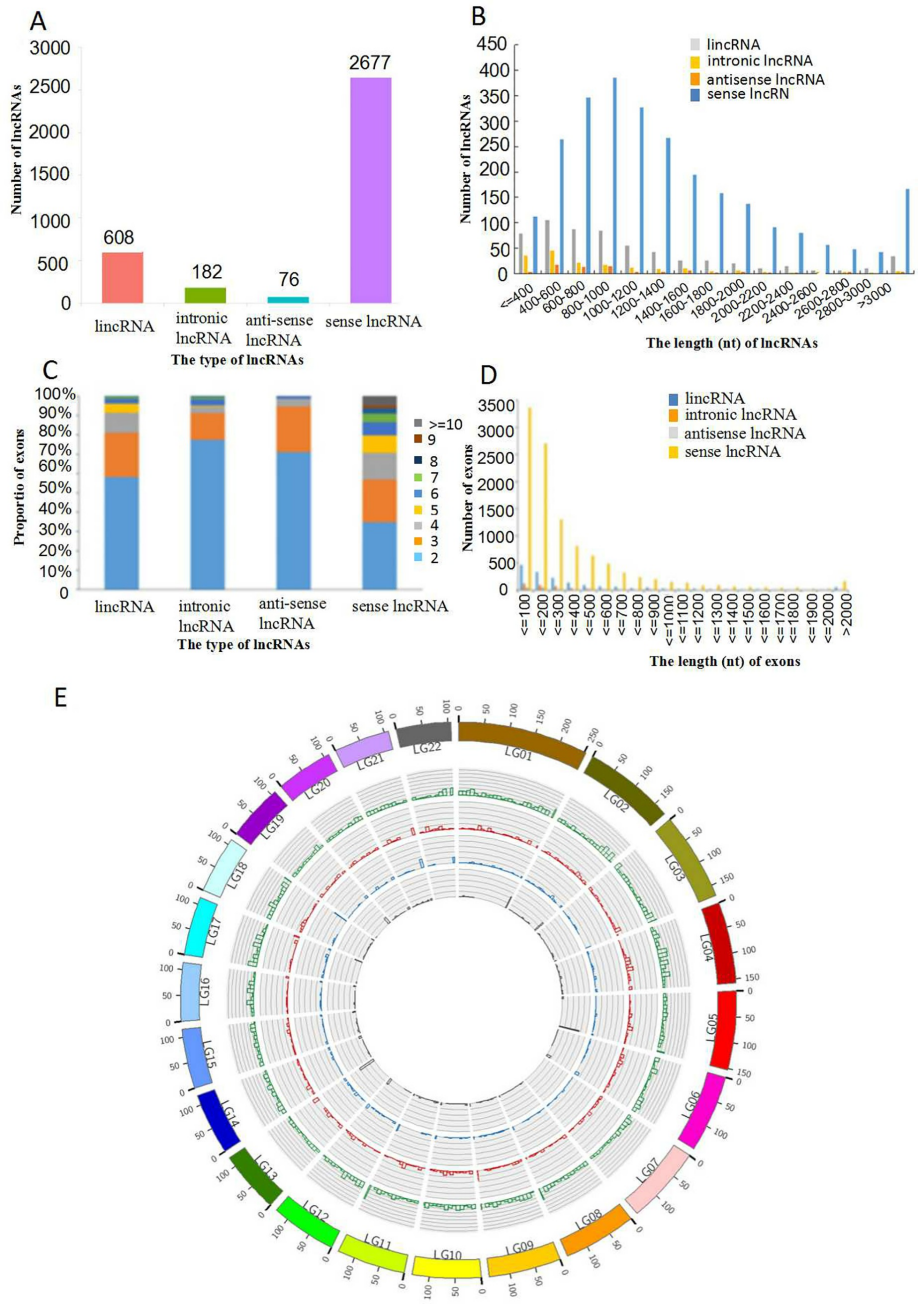
Description of the sequence characteristics of lncRNAs in *A*. *comosus* var. *bracteatus* leaves. (A) Numbers of lincRNAs, intronic lncRNAs, antisense lncRNAs and sense lncRNAs in *A*. *comosus* var. *bracteatus* leaves; (B) Comparison of the transcript length distributions of the four types of lncRNAs; (C) Analysis of the exon numbers of each transcript of the four types of lncRNAs; (D) Exon length analysis of the four types of lncRNAs; (E) Analysis of the lncRNAs along each chromosome, showing the number of lincRNAs in physical bins of 500 b for each chromosome. The circles from inside to outside represent antisense lncRNA (black), intronic lncRNA (blue), lincRNA (red) and sense lncRNA (green).

The 3,543 newly identified lncRNAs included lincRNAs, antisense lncRNAs, intronic lncRNAs and sense lncRNAs (Fig 3A). A majority of the lincRNAs and intronic lncRNAs were shorter than 1,000 nucleotides (Fig 3B). In addition, we also analyzed the number of exons in each lncRNA transcript. Most of the intronic lncRNAs (77.5%) and antisense lncRNAs (71.1%) contained two exons. Most of the lincRNAs (81.1%) contained two or three exons, and only a few sense lncRNAs (4.8%) contained more than 9 exons (Fig 3C). Among these lncRNAs, 27.8% of the lincRNAs and 27% of the intronic lncRNAs had exons shorter than 100 nucleotides, and most of the sense lncRNAs (55.2%) had exons shorter than 200 nucleotides. However, 4.4% of the antisense lncRNAs had exons longer than 2,000 nucleotides (Fig 3D). We analyzed the positional preferences of the lncRNAs on the chromosomes. The results showed that lincRNAs and sense lncRNAs were evenly distributed on every chromosome, indicating that there was no significant position preference for lincRNAs and sense lncRNAs. However, the intronic lncRNAs were unevenly distributed on LG11, LG18 and LG22, and the anti-lncRNAs were not distributed on LG7 and LG22 (Fig 3E).

### Conservation analysis of lncRNAs

**Fig 4:**
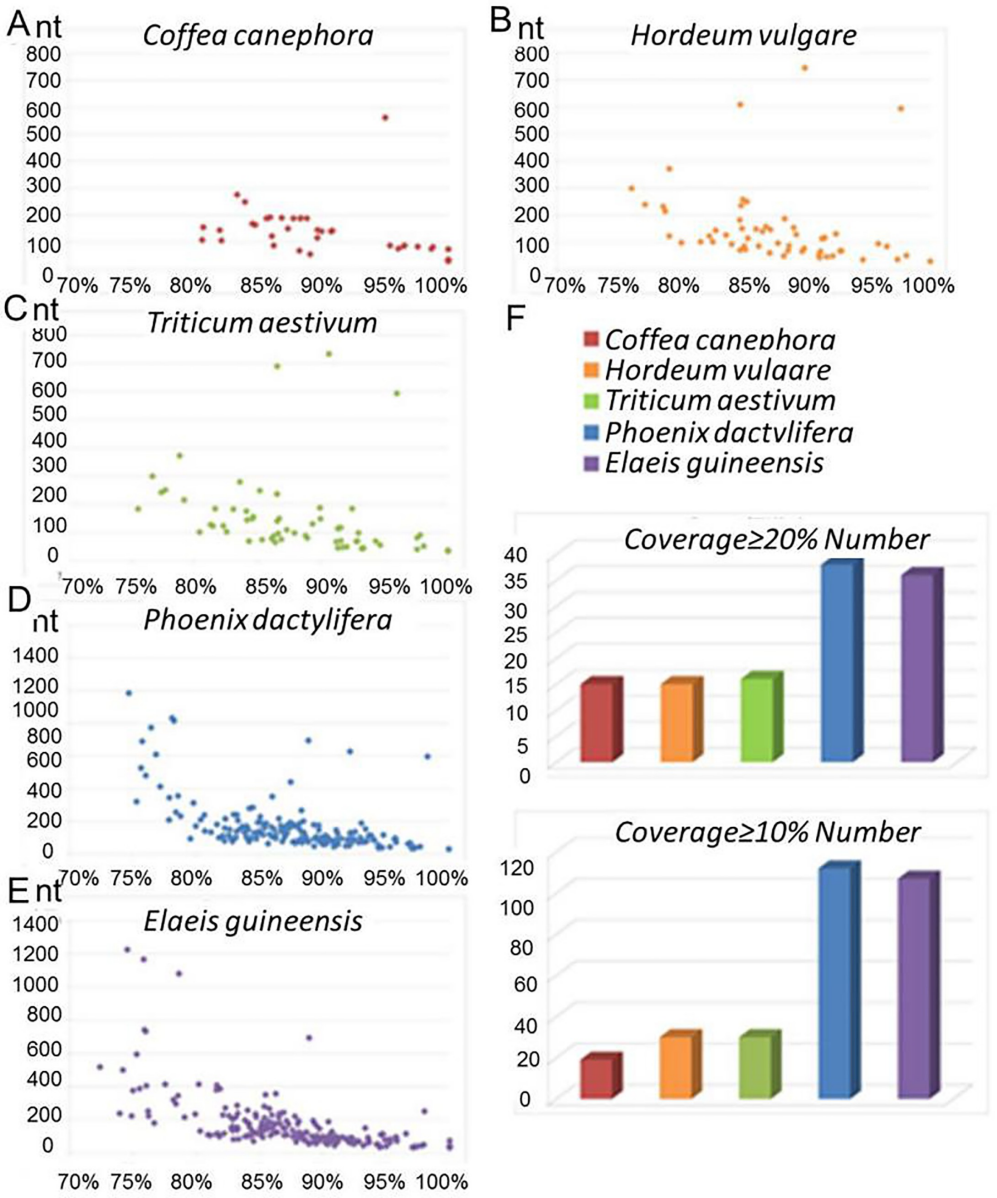
Conservation analyses of lncRNAs in *A*. *comosus* var. *bracteatus*. (A) *A*. *comosus* var. *bracteatus* lncRNAs that are conserved in *Coffea canephora*; (B) *A*. *comosus* var. *bracteatus* lncRNAs that are conserved in *Hordeum vulgare*; (C) *A*. *comosus* var. *bracteatus* lncRNAs that are conserved in *Triticum aestivum*; (D) *A*. *comosus* var. *bracteatus* lncRNAs that are conserved in *Phoenix dactylifera*; (E) *A*. *comosus* var. *bracteatus* lncRNAs that are conserved in *Elaeis guineensis*; (F) The number of conserved lncRNAs with more than 20% or 10% coverage regions.

To investigate the conservation of lncRNAs in *A*. *comosus* var. *bracteatus*, 3,543 lncRNA sequences were subjected to a BLAST search against the genome sequences of 5 representative plants (*Coffea canephora*, *Hordeum vulgare*, *Triticum aestivum*, *Phoenix dactylifera*, *Elaeis guineensis*) with a threshold E-value < 1e^-5^. These five plants, similar to *A*. *comosus* var. *bracteatus*, originate in the tropics and are monocots. Through bioinformatics analysis, we found that the sequences of these five plants have high homology with those of *A*. *comosus* var. *bracteatus*. A summary of the coverage values of the lncRNAs is shown in Fig 4. In the interspecies comparisons, we found that lncRNA conservation was lower than that of the protein-coding genes. Our results showed that 288 and 275 of lncRNAs were predicted to be conserved in *Phoenix* and *Elaeis*, respectively, and the length of the conserved sequences in *A*. *comosus* var. *bracteatu*s is longer than those in the other two species (Table 1). These results indicated that *A*. *comosus* var. *bracteatus* may exhibit higher lncRNA homology with these two species than with the other species. However, only 55 lncRNAs shared homology with the *Coffea* genome, while 97 and 99 lncRNAs were conserved in *Hordeum* and *Triticum*, respectively. A complete list of all the conserved lncRNAs can be found in S3 Table. In addition, the coverage values were also investigated to predict the species with the most homologous sequences to those of *A*. *comosus* var. *bracteatus*. The results suggested that *A*. *comosus* var. *bracteatus* lncRNAs were most highly conserved in *Phoenix*, followed by *Elaeis*.

**Table 1:** Summary of the *A*. *comosus* var. *bracteatus* lncRNAs that are conserved in *Coffea canephora*, *Hordeum vulgare*, *Triticum aestivum*, *Phoenix dactylifera*, and *Elaeis guineensis*.

Species	Total number	Length	Identity	Coverage > = 20% Number	Coverage > = 10% Number
*Coffea canephora*	55	29–562	80.56–100.00	15	19
*Elaeis guineensis*	275	31–1226	31–1226	36	107
*Hordeum vulgare*	97	31–744	76.00–100.0	15	30
*Phoenix dactylifera*	288	29–984	74.70–100.00	38	112
*Triticum aestivum*	99	34–734	75.54–100.00	16	30

### Comparison of the basic properties of lncRNAs and mRNAs

**Fig 5:**
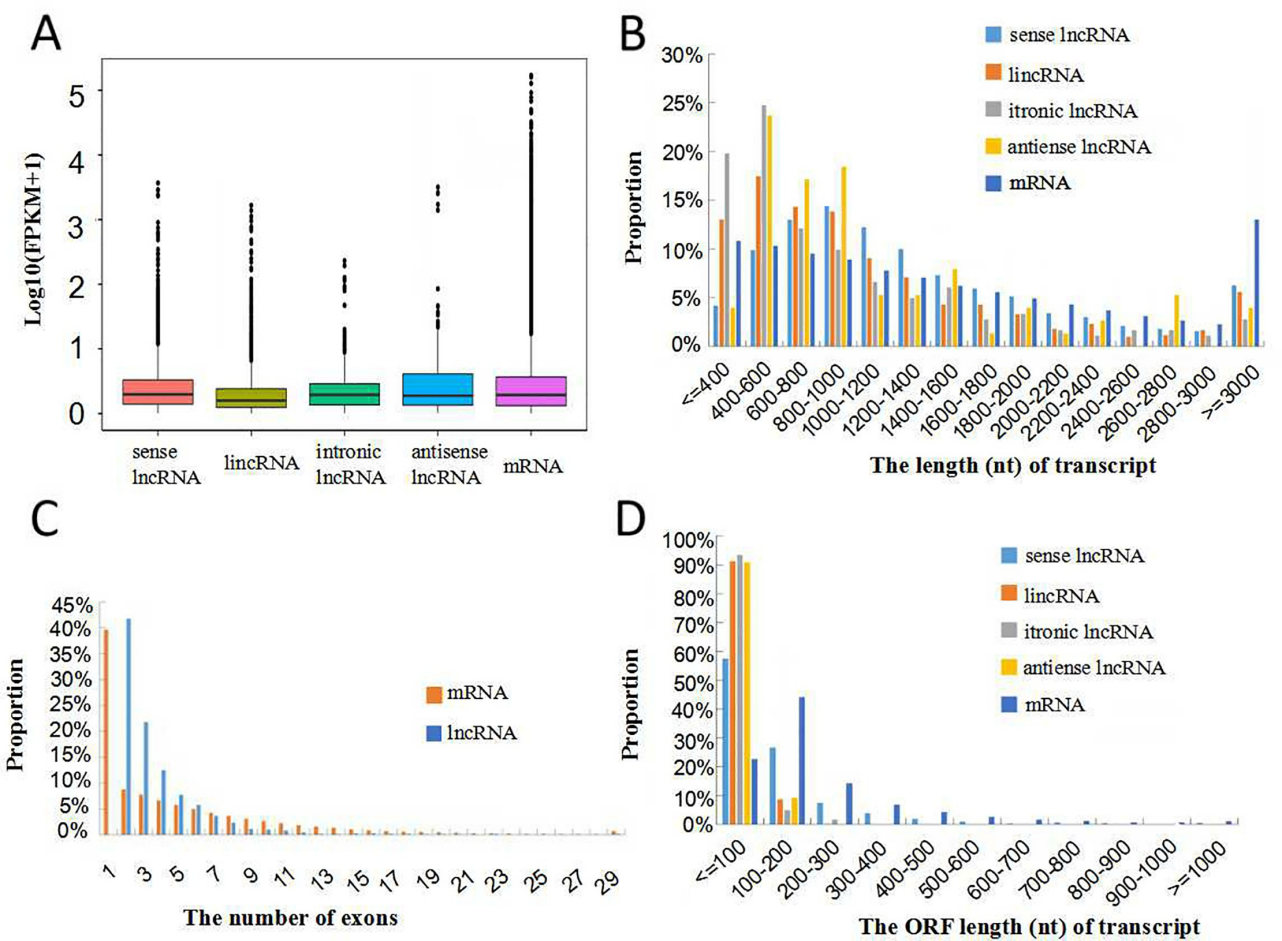
Comparison of the basic properties of lncRNAs and mRNAs. (A) Comparison of the expression levels of lncRNAs and mRNAs; (B) Comparison of the transcript lengths of lncRNAs and mRNAs; (C) Comparison of the number of exons in lncRNAs and mRNAs; (D) Comparison of the ORF lengths of lncRNAs and mRNAs.

To characterize the genomic features, four potential types of lncRNAs were separately compared with protein-coding mRNAs. Under the same conditions, the transcript abundances, lengths, exon numbers, and ORFs (open reading frames) of the lncRNAs and mRNAs in *A*. *comosus* var. *bracteatus* leaves were compared (Fig 5). The FPKM values showed that the abundances of intronic lncRNAs and lincRNAs were lower than those of mRNAs, the sense lncRNAs and mRNAs were expressed at similar levels, and the abundances of antisense lncRNAs was slightly higher than those of mRNAs, indicating that most lncRNAs were less extensively transcribed (Fig 5A). In addition, we found that the lengths of sense lncRNAs were longer than those of mRNAs in the range from 600 to 2000, while intronic lncRNAs were longer than mRNAs in the ranges 400–600, 800–1000 and below 400. However, mRNAs were longer than lncRNAs from 1600 to 3000, and above 3000, antisense lncRNAs were generally shorter than mRNAs (Fig 5B). Moreover, the number of exons present in the lncRNAs was also lower than that in mRNAs. Most lncRNAs (76.04%) had fewer than 5 exons, and 41.80% of the lncRNAs had two exons; in contrast, 31.62% of the mRNAs had more than 5 exons, and the exons exhibited a wider range than they did in the lncRNAs (Fig 5C). Most lncRNA ORFs were shorter than those of the mRNAs, while most (77.35%) of the mRNAs had ORFs greater than 100 nucleotides (Fig 5D).

### New alternative splicing event prediction

**Fig 6:**
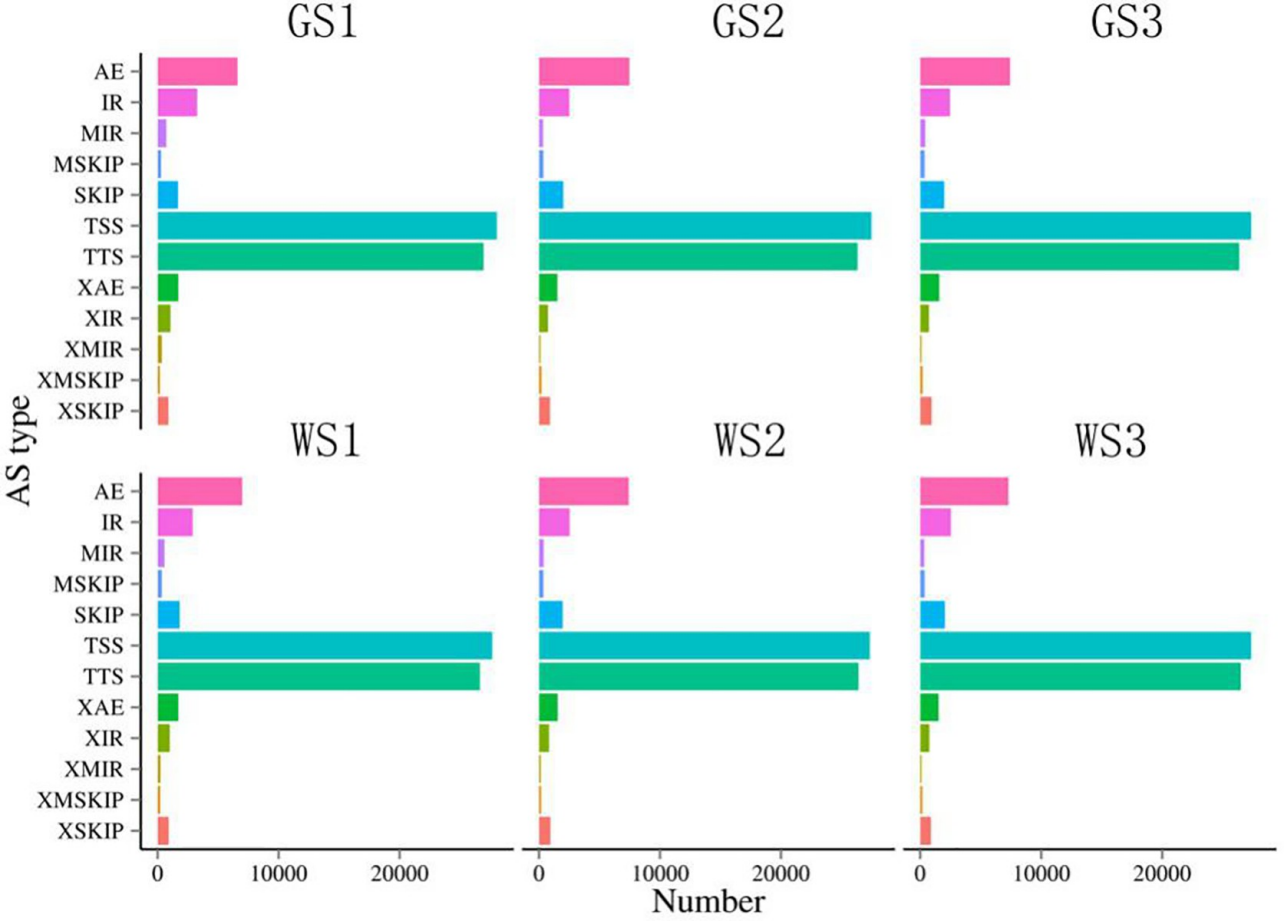
Six samples examined for splicing event statistics. (1) TSS: alternative first 5' exon. (2) TTS: alternative last 3' exon; (3) SKIP: skipped exon; (4) XSKIP: approximate SKIP; (5) MSKIP: multiexon SKIP; (6) XMSKIP: approximate MSKIP; (7) IR: intron retention; (8) XIR: approximate IR; (9) MIR: multi-IR; (10) XMIR: approximate MIR; (11) AE: alternative exon ends; (12) XAE: approximate AE. X axis: The number of transcripts of a variable splice. Y axis: 12 variable shear types.

Based on RNA-seq data, we studied alternative splicing events in *A*. *comosus* var. *bracteatus*. The splicing events were divided into twelve types: (1) TSS: substituted for the first 5' exon; (2) TTS: substituted for the last 3' exon; (3) SKIP: Skipped exon; (5) MSKIP: multiple explicit SKIP; (6) XMSKIP: approximate MSKIP; (7) IR: intron retention; (8) XIR: approximate IR; (11) MIR: multiple IR; (10) XMIR: approximate MIR; (11) AE: substituted exon ends (5', 3' or both); and (12) XAE: approximate AE. The distributions of the identified splicing events among the 12 types are shown in Fig 6. The distributions of the identified splicing events among the 12 types were similar between the green and white leaves at three developmental stages. The number of each type is quite different. Among them, the majority of AS events were TSS (35.35%) and TTS (34.06%). In contrast, XMIR and XMSKIP accounted for only 0.44% and 0.46% of the total AS events (S4 Table).

### Identification of chlorophyll-metabolism-related lncRNAs

**Fig 7:**
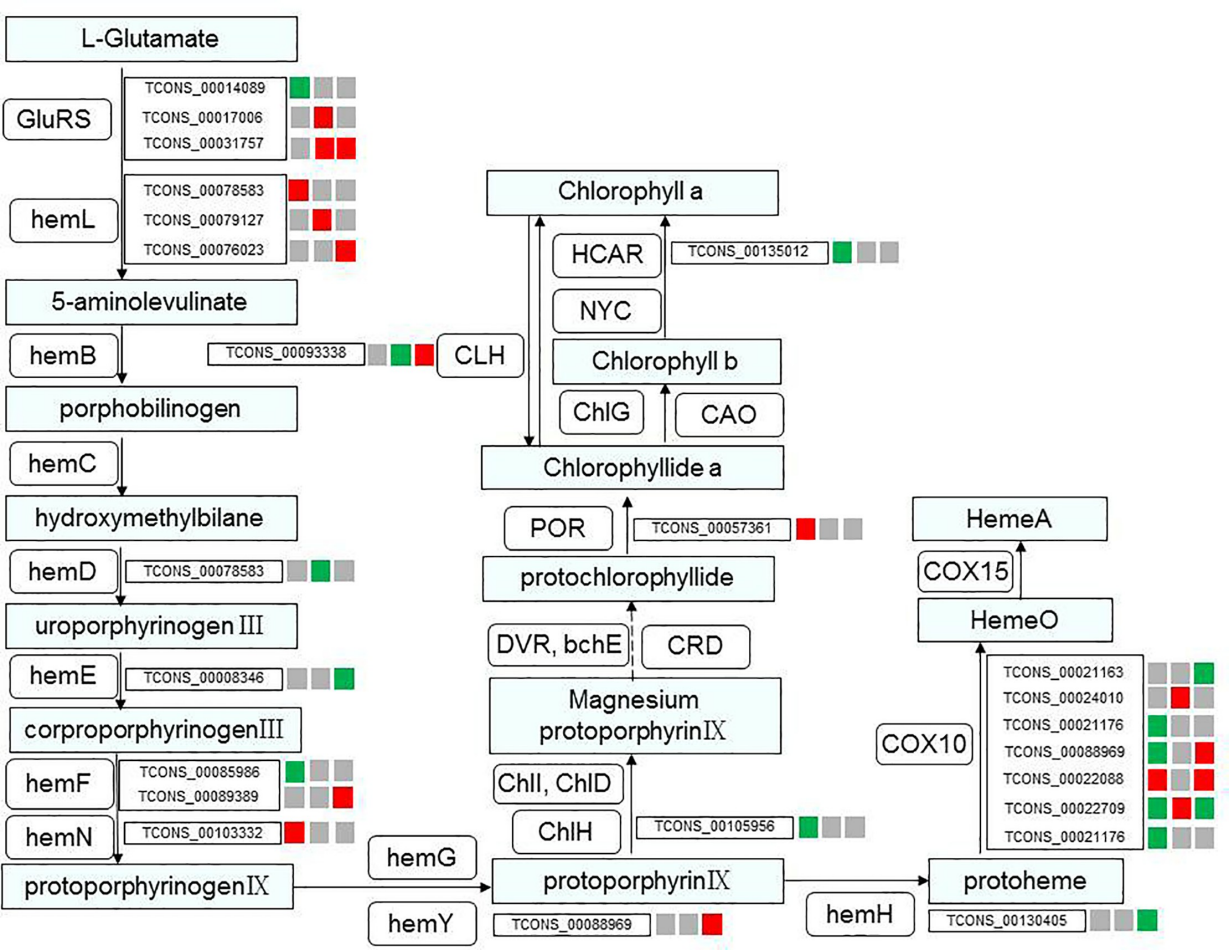
Porphyrin and chlorophyll metabolism in *A*. *comosus* var. *bracteatus*. The expression patterns of lncRNAs with target genes related to chlorophyll biosynthesis. The three small squares next to the lncRNA names indicate the expression patterns of the lncRNAs at the three developmental stages. Red indicates upregulation in CWh leaves; green indicates downregulation in CWh leaves; and gray indicates no significant difference between CGr and CWh leaves.

LncRNA target genes related to chlorophyll biosynthesis were chosen to analyze the mechanism of lncRNA-mediated regulation of the albinism of white leaf cells. To reveal the cooperation between lncRNAs and mRNAs, chlorophyll-biosynthesis-related lncRNAs and their target genes were chosen to construct a lncRNA-mRNA interaction network (Fig 5). In this interaction network, 36 target mRNAs related to chlorophyll metabolism were predicted to interact with 86 lncRNAs (S1 Fig). Most mRNAs are targeted by several lncRNAs, but some mRNAs are targeted by only one lncRNA. For example, heme O synthase (COX10) is targeted by seven lncRNAs, but magnesium chelatase subunit H (ChlH) is targeted by one lncRNA. Protochlorophyllide oxidoreductase (POR) is also targeted by only one lncRNA. COX10 is found in many archaea, bacteria, and eukaryotes and produces heme O, which, in many cases, is further modified into heme A. In organisms that produce heme A, the COX10 enzyme forms a complex with heme A synthase [44, 45]. ChlH showed a negative regulatory relationship with lncRNAs; it controls the first committed step of chlorophyll biosynthesis and is a branch point for the two major routes in the tetrapyrrole pathway [46,47]. The significantly differentially expressed lncRNAs (| log2 (FC) | ≥ 1.5 and FDR ≤ 0.05) between the CGr and CWh leaves were also selected and analyzed (S5 Table). Twenty-five significantly differentially expressed lncRNAs putatively interacted with 16 target mRNAs related to chlorophyll metabolism. These lncRNAs and mRNAs may play important roles in the chlorophyll biosynthesis of *A*. *comosus* var. *bracteatus* leaves. Notably, *TCONS_00022709*, which targets the heme O synthase (COX10) gene, is differentially expressed between CGr and CWh leaves at the three developmental stages (Fig 7).

### LncRNAs related to photosynthesis

According to previous studies, the top three enriched pathways of DEGs between the CGr and CWh leaves of *A*. *comosus* var. *bracteatus* are photosynthesis, porphyrin and chlorophyll metabolism, and carotenoid biosynthesis [28]. Thus, significantly differentially expressed lncRNA target genes possibly related to photosynthesis were analyzed. A total of 31 lncRNAs were found to be differentially expressed between CGr and CWh leaves at the three developmental stages. Among the targets, PsaE and Psb28 were targeted by the same lncRNAs. PsbB and PasbH were targeted by the lncRNA *TCONS_00103730*. ATPF0C and ATPF0A were targeted by the lncRNA *TCONS_00061869*. The number of lncRNA target genes with functions in the photosynthetic system were as follows: PS I (9), PS II (6), photosynthetic electron transport (2), ATP synthase (5), light-harvesting chlorophyll protein complex (LHC) (13) (S6 Table). This finding suggested that photosynthesis is regulated by lncRNAs in a complex manner.

### Expression patterns of eight lncRNAs and their potential target genes

**Fig 8:**
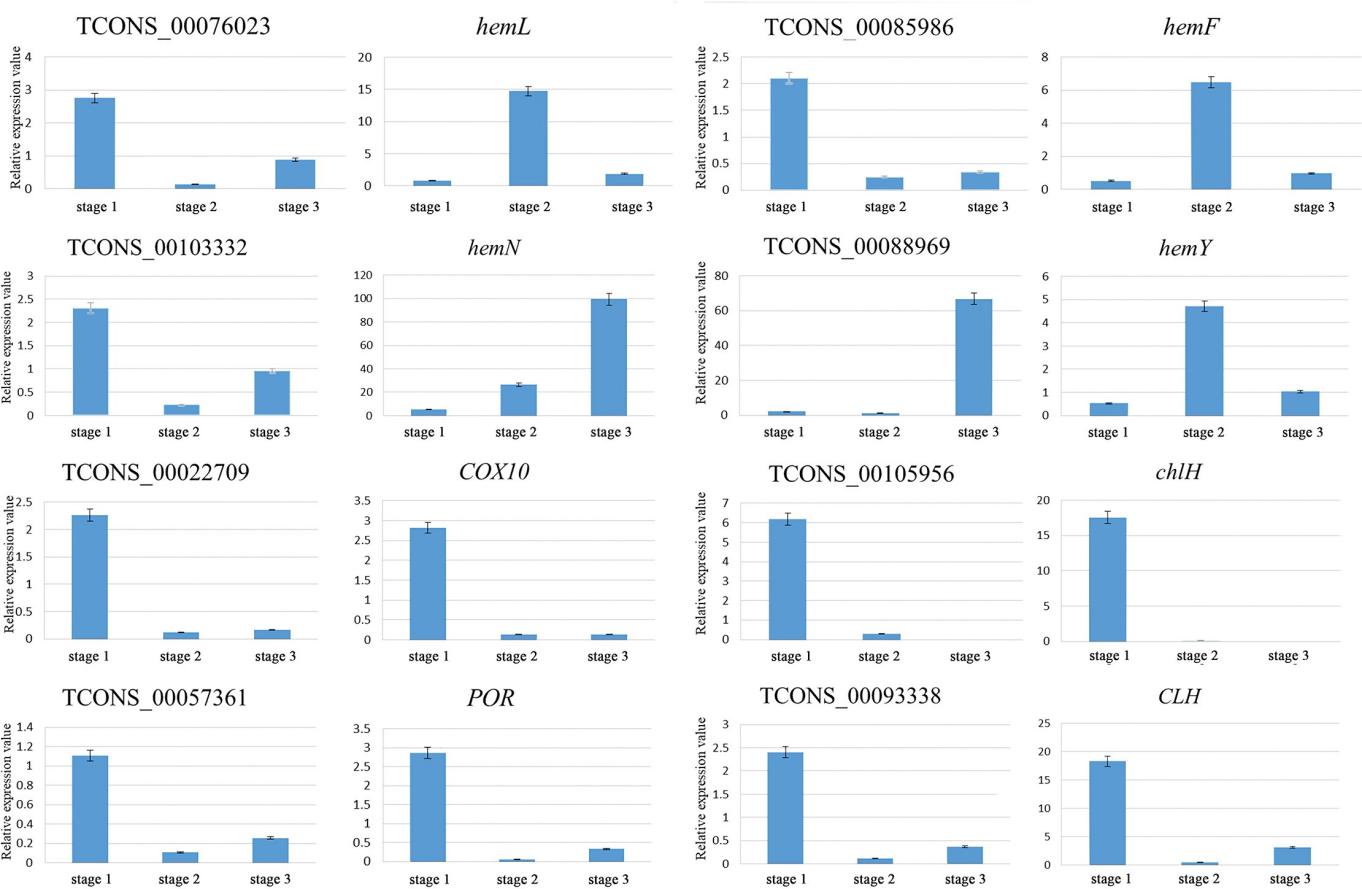
qRT-PCR-based validation of putative lncRNAs and their potential target genes in *A*. *comosus* var. *bracteatus*. Expression profiles of 8 putative lncRNAs and their potential target genes in CGr and CWh leaves at the three developmental stages. The relative expression values were the CWh/CGr values. Each bar represents the mean value from triplicate experiments ± SD.

To confirm the potential mechanism of regulation of target genes by lncRNAs, qRT-PCR was used to measure the expression profiles of eight putative lncRNAs and their potential target genes related to chlorophyll biosynthesis (Fig 8). Eight putative lncRNAs associated with chlorophyll metabolism were selected for quantitative PCR-based validation. The results showed that four lncRNAs and their target genes showed the opposite trend, and the other four lncRNAs and their target genes showed the same trend.

## Discussion

High-throughput sequencing technology has shown that only a small fraction of the eukaryotic genome sequence that is generally transcribed belongs to protein-coding genes, and many noncoding RNAs have been identified as important regulators of biological processes [48]. A large number of lncRNAs have been found to be transcribed in plant genomes [49]. The functions of lncRNAs may be related to growth and development, plant photomorphogenesis, abiotic and biological stress reactions and other biological processes [50–52]. In this study, we present the first comprehensive analysis of lncRNAs in *A*. *comosus* var. *bracteatus*. These data provide useful resources for future functional genomics research and the study of epigenetic regulation mechanisms in *Bromelia*.

The lncRNAs identified in *A*. *comosus* var. *bracteatus* are not well conserved and have fewer exons than do the protein-coding genes. The transcript levels of the lncRNAs were also significantly lower than those of the mRNAs, consistent with the results of studies on *Arabidopsis* and rice [53,54]. In this study, we removed candidate transcripts with only 1 exon, because transcripts with one or more exons are more stable than unspliced (single-exon) transcripts. In general, lncRNAs are not conserved between plant species, probably because the primary model for rapid lncRNA emergence and decay in plants involves frequent whole genome duplications and genome rearrangements, which are more often seen in plants than in vertebrates. Thus, polyploidy is a major driver of genome evolution in monocots, including this system, as in most other plant lineages.

Alternative splicing of pre-mRNA represents a major mechanism underlying increased transcriptomic and proteomic complexity. LncRNAs participate in transcriptional and posttranscriptional regulation of gene expression via a variety of complex mechanisms [49]. LncRNAs have been reported to regulate growth and development in plants [55]. It has been reported that lncRNAs can hijack nuclear alternative splicing regulators to modulate alternative splicing patterns during development [56]. The *Arabidopsis* ASCO lncRNA (alternative splicing competitor lncRNA) regulates plant root development by binding to regulators of alternative splicing, namely, nuclear speckle RNA-binding proteins, and then hijacking these proteins to change the patterns of alternative splicing to produce alternatively spliced isoforms [56,57]. In some plants, IR is predominant, such as maize, sorghum and alfalfa [58–60]. However, IR accounted for only 6.87% of alternative splicing events, and TSS and TTS were much more numerous than the other types in *A*. *comosus* var. *bracteatus*. One possible reason is the large differences between species. Future investigation of alternative splicing in *A*. *comosus* var. *bracteatus* will continue with the integrative analysis of lncRNA, miRNA, mRNA and proteins.

It has been reported that the biosynthesis and accumulation of pigments in plant leaves are regulated by various internal and external factors, and changes in Chl concentration lead to changes in leaf color [61,62]. In rice (C3) and maize (C4) seedling growth, lncRNAs, as potential targets of miRNAs, may serve as regulators of photosynthesis [63]. The insertion of a lncRNA into the promoter of the *BnaA07*.*HO1* gene downregulated the expression of *BnaA07*.*HO1*, which resulted in yellow-green seedling leaves [64]. Previous studies have confirmed that the loss of green color is caused by the inhibition of the expression of genes involved in Chl biosynthesis or chloroplast development, such as magnesium chelatase subunit (ChlH), uroporphyrinogen decarboxylase 1 (HemE) and protochlorophyllide oxidoreductase (POR) [65–69]. In this study, 25 significantly differentially expressed lncRNAs were identified with possible target gene functions in chlorophyll biosynthesis. Among them, some of the differentially expressed genes played key roles and were targeted by one or more significantly differentially expressed lncRNAs. For instance, the lncRNA *TCONS_00093338*, targeting chlorophyllase, was significantly differentially expressed between the CGr and CWh leaves during the three developmental stages. Chlorophyllase participates in degreening processes and adjusts the turnover and homeostasis of chlorophyll [69]. ChlH, which conducts the first committed step of chlorophyll biosynthesis and is a branch point for two major routes in the tetrapyrrole pathway [45,46,70], is a target that presents a positive correlation with the differentially expressed lncRNA *TCONS_00105956*. The lncRNA *TCONS_00057361* is predicted to target the POR gene and exhibits a positive regulatory relationship. Catalysis occurs via a light-dependent trans-reduction of the D-ring of protochlorophyllide (PChlide, P) to produce chlorophyllide (Chlide, C). This process plays an important role in the synthesis of chlorophyll in higher plants [71–73]. The lncRNA *TCONS_00022709*, targeting COX10, is significantly differentially expressed between CGr and CWh leaves during leaf development. These results suggest that these lncRNAs could be important in leaf albinism and development and are worth studying further in the future.

Total or partial loss of photosynthetic pigments is an important reason for albinism in plants [74]. The albinism of leaves significantly affects photosynthesis. In this study, we discovered 31 significantly differentially expressed lncRNAs that target 20 photosynthesis-related mRNAs. Differentially expressed lncRNAs mainly target genes encoding the light-harvesting chlorophyll protein complex PSII and F-type ATPase. Only the lncRNAs *TCONS_00015572* and *TCONS_00016339*, targeting LHCA4 (light-harvesting complex I chlorophyll a/b binding protein 4), were significantly differentially expressed between CGr and CWh leaves during the three developmental stages. The lncRNA *TCONS_00015572* and LHCA4 exhibited a positive regulatory relationship, and the lncRNA *TCONS_00016339* and LHCA4 exhibited negative regulation in the third developmental stage of CGr and CWh leaves. These results indicated the complex regulatory mechanisms and various functions associated with lncRNAs in *A*. *comosus* var. *bracteatus*.

## Conclusions

In this study, we report the first genome-wide lncRNA profiling of *A*. *comosus var*. *bracteatus*. In total, 3,543 lncRNAs were identified, among which 1,451 were differentially expressed between CGr and CWh leaves. LncRNAs predicted to possibly target genes related to chlorophyll biosynthesis and photosynthesis were analyzed to reveal the lncRNA-mediated mechanisms of albinism regulation in white leaf cells. *TCONS_00105956*, *TCONS_00057361*, *TCONS_00022709*, and other lncRNAs may be key epigenetic regulators of leaf albinism in *A*. *comosus* var. *bracteatus*. These results will serve as a valuable basis for further research on the epigenetic regulatory mechanisms of *A*. *comosus var*. *bracteatus*. In subsequent experiments, the function of these key lncRNAs will be verified by transformation. And the tissue-specific expression vectors will be constructed to regulate the expression of these key lncRNAs to regulate the chlorophyll content in the leaves to adjust the leaf color and enhance the ratio of chimeric plants during tissue culture. Furthermore, synergistic regulation of the expression of lncRNAs function in anthocyanins, carotenoids and chlorophyll biosynthesis can obtain new genotypes with different leaf colors, and promote the breeding of *A*. *comosus* var. *bracteatus*.

## Supporting information

S1 Fig LncRNA-mRNA interaction network associated with chlorophyll metabolism in *A. comosus* var. *bracteatus* leaves. Thirty-six mRNAs interacted with 86 lncRNAs. Genes shown with red triangles are lncRNAs, and genes shown with green circles are mRNAs.
(PDF)

S1 Table Primers for qPCR analysis. (DOCX)

S2 Table The sequences of all 3,543 lncRNAs identified by CPC, CNCI, CPAT, and Pfam. (XLSX)

S3 Table Conservation blast results of all 1,440 lncRNAs. (XLSX)

S4 Table The numbers of new alternative splicing events in *A. comosus* var. *bracteatus*. (XLSX)

S5 Table The significantly differentially expressed lncRNAs related to chlorophyll biosynthesis between the CGr and CWh leaves. (XLSX)

S6 Table The significantly differentially expressed lncRNAs related to photosynthesis between the CGr and CWh leaves. (XLSX)
