# Supplementary material for: Screening and characterization of long noncoding RNAs involved in the albinism of Ananas comosus var. bracteatus leaves
Source: PLoS One. 2019 Nov 22;14(11):e0225602. doi: 10.1371/journal.pone.0225602 (PMC6874346; doi:10.1371/journal.pone.0225602)
Supplement: S1 Table — (DOCX) [file pone.0225602.s002.docx]

**Screening and characterization of long noncoding RNAs involved in the albinism of *A. comosus* var. *bracteatus* leaves**

**Zhen Lin^1^,** **Yingyuan Xiong^1^, Yanbin Xue^1^, Meiqin Mao^1^,Yixuan Xiang^1^, Yehua He^2^, Fatima Rafique^1^, HaoHu^1^, Jiawen Liu^1^, Xi Li^1^, Lingxia Sun^1^, Mingdong Ma^1^, Zhuo Huang^1^, Jun Ma^1^***

^1^ College of Landscape Architecture of Sichuan Agricultural University, Chengdu, Sichuan 611100, China.

^2^ Horticultural Biotechnology College of South China Agricultural University, Guangzhou, Guangdong 510642, China.

*Corresponding author; E-mail: junma365@hotmail.com

Tel/Fax: +86−28−82652812.

**S1 Table. The qRT-PCR primers used in this study**

| Name | Primer sequence (5'-3') |
| --- | --- |
| TCONS_00076023 | F: CGAGCAGAGTAATAAACAGGGTAA |
|  | R: TGGTATTGGGTGTGTAGAAATGA |
| TCONS_00085986 | F: GATGGTGGTTTTGGATTGGTG |
|  | R: TTGTTGGAGAGAGATTTTACTGTGT |
| TCONS_00103332 | F: TTTGCGAGTTCTTTATGTGCC |
|  | R: TGCTTCAGTTCTTGGACGGT |
| TCONS_00088969 | F: AAAATACGCACGCCTCCTG |
|  | R: GCTTCTGCCTCCCATCTACAA |
| TCONS_00022709 | F: CGTATGCAGCTATTAAGTTAATCTCA |
|  | R: GAATGTATTTCATGGGTCCCAA |
| TCONS_00105956 | F: AAGCCTTCTCACGAGTTTGGT |
|  | R: CCTTTTTCACTGTAGGACGACA |
| TCONS_00057361 | F: GTGAAGGAGTCCGATTAGCAA |
|  | R: CGCCGTGGATTAGAGGGT |
| TCONS_00093338 | F: TTAGACGATTTTTTGTGCTTCC |
|  | R: GCGATGTTGTTACTTGTGCG |
| glutamate-1-semialdehyde 2,1-aminomutase (hemL) | F: CCAGCCCATCGTTTTTGACT |
|  | R: ATAGCAGGACCCCAGGAACC |
| coproporphyrinogen III oxidase (hemF) | F: TGGTGGTTTGGAGGTGGGAC |
|  | R:CTTGAATCTGGGGTAGAAACTTGGA |
| oxygen-independent coproporphyrinogen III oxidase (hemN) | R:CGAGCAGGGTTGTAGACATCAGTG |
|  | F: GGCGTATCTCAGCATTCTTGGGT |
| protoporphyrinogen/coproporphyrinogen III oxidase (hemY) | R: GGGTTCGGGCAGTTACATC |
|  | F: CCCAGGAGGAGCACGATTAG |
| heme o synthase (COX10) | R: TGGCTTGGAAGGCTAA |
|  | F: AGAAGTGGAGGAATGG |
| magnesium chelatase subunit H (ChlH) | F: GGACAACGAACAAGA |
|  | R: CTCCCAAGAACTCAA |
| protochlorophyllide reductase (POR) | F: CAAGGACAGCAAGGTG |
|  | R: GGGAGGGAACAGGAGG |
| Chlorophyllase (CLH) | F: TCTTCCCCCCTTGTGC |
|  | R: TCCCTTGCTTTGCCAT |
| 18S | F: ATGGTGGTGACGGGTGAC |
|  | R: CAGACACTAAAGCGCCCGGTA |
| α-tubulin | F: CCATACAATAGCGTCCTA |
|  | R: ATAGCCTCGTTATCCAATA |
