# Supplementary material for: Screening and characterization of long noncoding RNAs involved in the albinism of Ananas comosus var. bracteatus leaves
Source: PLoS One. 2019 Nov 22;14(11):e0225602. doi: 10.1371/journal.pone.0225602 (PMC6874346; doi:10.1371/journal.pone.0225602)
Supplement: S1 Fig — Thirty-six mRNAs interacted with 86 lncRNAs. Genes shown with red triangles are lncRNAs, and genes shown with green circles are mRNAs. (PDF) [file pone.0225602.s001.pdf]

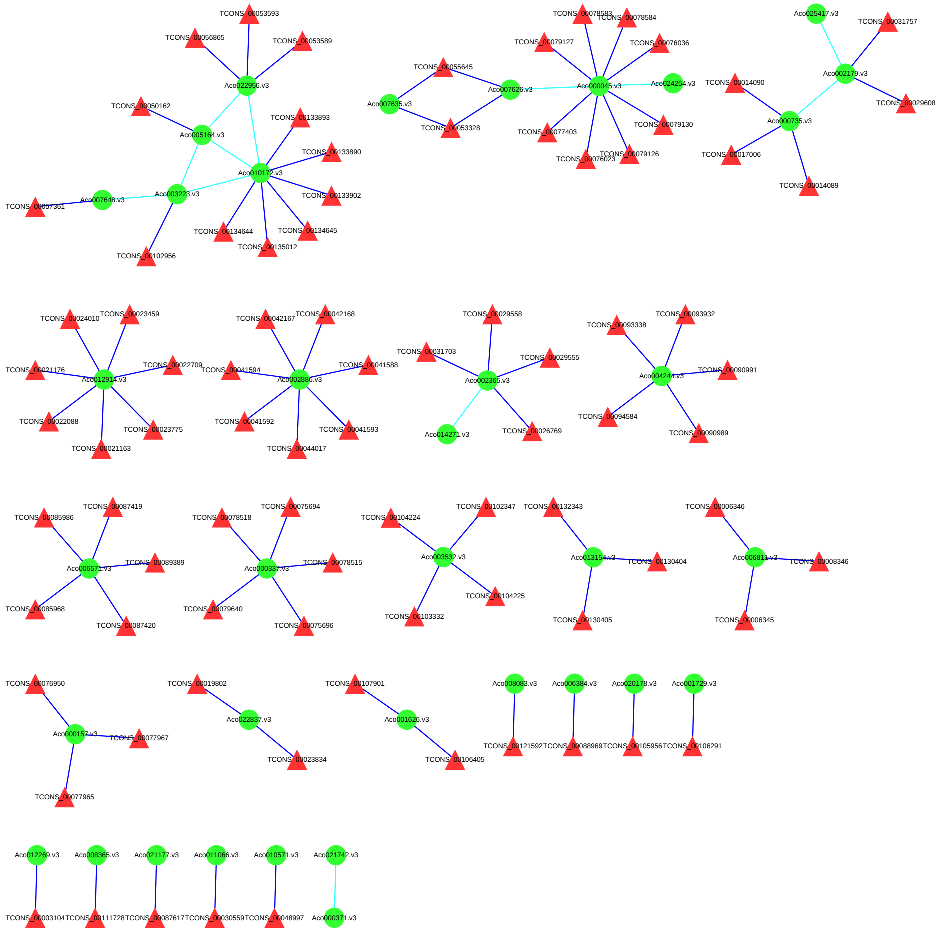

TCONS_00053593
TCONS_00056865
TCONS_00053589
Aco022956.v3
TCONS_00050162
Aco005164.v3
TCONS_00133893
TCONS_00133890
Aco010172.v3
TCONS_00133902
Aco007648.v3
Aco003223.v3
TCONS_00057361
TCONS_00134644
TCONS_00134645
TCONS_00135012
TCONS_00102956
TCONS_00055645
Aco007635.v3
Aco007626.v3
TCONS_00053328
TCONS_00078583
TCONS_00078584
TCONS_00079127
TCONS_00076036
Aco000045.v3
Aco024254.v3
TCONS_00077403
TCONS_00079130
TCONS_00076023
TCONS_00079126
Aco025417.v3
TCONS_00031757
Aco002179.v3
TCONS_00014090
TCONS_00029608
Aco000735.v3
TCONS_00017006
TCONS_00014089
TCONS_00024010
TCONS_00023459
TCONS_00021176
TCONS_00022709
Aco012914.v3
TCONS_00022088
TCONS_00023775
TCONS_00021163
TCONS_00042167
TCONS_00042168
TCONS_00041594
TCONS_00041588
Aco002886.v3
TCONS_00041592
TCONS_00041593
TCONS_00044017
TCONS_00029558
TCONS_00031703
TCONS_00029555
Aco002365.v3
Aco014271.v3
TCONS_00026769
TCONS_00093338
TCONS_00093932
Aco004244.v3
TCONS_00090991
TCONS_00094584
TCONS_00090989
TCONS_00085986
TCONS_00087419
Aco006571.v3
TCONS_00089389
TCONS_00085968
TCONS_00087420
TCONS_00078518
TCONS_00075694
Aco000337.v3
TCONS_00078515
TCONS_00079640
TCONS_00075696
TCONS_00104224
TCONS_00102347
Aco003532.v3
TCONS_00104225
TCONS_00103332
TCONS_00132343
Aco013154.v3
TCONS_00130404
TCONS_00130405
TCONS_00006346
Aco006811.v3
TCONS_00008346
TCONS_00006345
TCONS_00076950
Aco000157.v3
TCONS_00077967
TCONS_00077965
TCONS_00019802
Aco022837.v3
TCONS_00023834
TCONS_00107901
Aco001626.v3
TCONS_00106405
Aco008083.v3
Aco006384.v3
Aco020178.v3
Aco001729.v3
TCONS_00121592
TCONS_00088969
TCONS_00105956
TCONS_00106291
Aco012269.v3
Aco008365.v3
Aco021177.v3
Aco011066.v3
Aco010571.v3
Aco021742.v3
TCONS_00003104
TCONS_00111728
TCONS_00087617
TCONS_00030559
TCONS_00048997
Aco000371.v3
